# The Health Belief Model Applied to COVID-19 Vaccine Hesitancy: A Systematic Review

**DOI:** 10.3390/vaccines10060973

**Published:** 2022-06-18

**Authors:** Yam B. Limbu, Rajesh K. Gautam, Long Pham

**Affiliations:** 1 Feliciano School of Business, Montclair State University, 1 Normal Ave, Montclair, NJ 07043, USA; 2 Department of Anthropology, Dr. Harisingh Gour Central University, Sagar 470003, India; goutamraj2006@gmail.com; 3 Department of Decision Sciences and Economics, College of Business, Texas A&M University at Corpus Christi, 6300 Ocean Drive, Corpus Christi, TX 78412, USA; lpham11@islander.tamucc.edu

**Keywords:** health belief model, COVID-19, vaccine hesitancy, systematic review, perceived severity, perceived susceptibility, perceived benefits, perceived barriers, cues to action, self-efficacy

## Abstract

This study systematically analyzes the research that used the Health Belief Model (HBM) as a theoretical basis to examine the influence of HBM constructs on COVID-19 vaccine hesitancy. Following PRISMA guidelines, PubMed, Web of Science, Google Scholar, and Scopus were searched for quantitative studies. Sixteen studies with 30,242 participants met inclusion criteria. The prevalence of COVID-19 vaccine hesitancy was 33.23% (95% CI 24.71–41.39%). Perceived barriers and perceived benefits were the most common HBM constructs that were significantly associated with vaccine hesitancy. While perceived benefits was inversely associated, a positive association was found between perceived barriers and vaccine hesitancy. Other HBM constructs that were frequently examined and inversely associated were perceived susceptibility, cues to action, perceived severity, and self-efficacy. The most common HBM modifying factor that was directly associated with COVID-19 vaccine hesitancy was gender, followed by education, age, geographical locations, occupation, income, employment, marital status, race, and ethnicity; however, a few studies report inconsistent results. Other modifying variables that influenced vaccine hesitancy were knowledge of COVID-19, prior diagnosis of COVID-19, history of flu vaccination, religion, nationality, and political affiliation. The results show that HBM is useful in predicting COVID-19 vaccine hesitancy.

## 1. Introduction

The outbreak of COVID-19 has severely affected the world with devastating consequences. As of 10 June 2022, there have been over 532 million confirmed cases of COVID-19 globally, and over 6.3 million deaths [1]. COVID-19 continues to have an unprecedented effect on lives, livelihoods, economies, and so on. Several potential vaccines have been developed and nine are approved by the EUA and different countries, and three are approved for use in the United States [2]. Despite this success and the availability of vaccines, government and business mandates, and public education campaigns that have convinced some people to accept the vaccination against COVID-19; however, this remains a major challenge. As a result, many people are still hesitant to be vaccinated against COVID-19 or less inclined to receive booster shots, or even less likely to vaccinate their offspring. Several countries, including some African countries, have not yet achieved herd immunity [3]. Morens et al. [4] indicate that there are significant obstacles to achieving complete herd immunity with COVID-19. Herd immunity occurs when a large portion of a community or population becomes immune to a disease or infection, either through vaccination or due to a previous infection [4]. Thus, herd immunity can only be achieved with mass vaccination.

Still, a significant portion of the global population is unvaccinated or hesitant to vaccinate against COVID-19. While 78% of the population had received at least one vaccine dose, 66% of people were fully vaccinated, and 46% of those fully vaccinated had received a booster or an additional dose as of 2 May 2022 [5]. The pace of vaccinations continues to slow in the U.S. Even after more than a year of COVID vaccine drives, still, a substantial portion of the world’s population has not received even a single dose. The vaccination rate is still low in many developing countries, especially in Africa, and is still far from achieving herd immunity. Thus, achieving herd immunity against SARS-CoV-2 is becoming difficult, due to a combination of factors that include features of the virus as well as current societal dynamics [4]. The COVID vaccines, including boosters, are proven to be safe and effective at preventing infection or reducing the risk of serious effects of the virus. The acceptance of vaccination is crucial to achieving herd immunity across different populations ending the pandemic or transitioning into an endemic. Convincing vaccine-hesitant populations to get vaccinated against COVID-19 is difficult [6].

Vaccine hesitancy refers to a delay in acceptance or refusal of safe vaccines despite the availability of vaccination services [7]. Various factors can influence vaccine hesitancy including socio-economic, psychological, and informational aspects. People’s health beliefs are major determinants of COVID-19 vaccine hesitancy. The Health Belief Model (HBM) is one of the most widely used models for understanding vaccination behavior against COVID-19. The theory holds that health-related behavior depends on the combination of several factors, namely, perceived susceptibility, perceived severity, perceived benefits, perceived barriers, cues to action, and self-efficacy [8].

Yet, no systematic review has addressed the application of HBM in predicting COVID-19 vaccine hesitancy. Thus, this is the first systematic review to explore the prevalence of COVID-19 vaccine hesitancy and understand the key HBM constructs that were significantly associated with COVID-19 vaccine hesitancy.

This study will provide important insights to drive vaccinations and public health interventions. Specifically, this study will enrich the understanding of the health belief-related barriers and facilitators affecting COVID-19 vaccine hesitancy. As acceptance of the vaccine among people is driven by their perceptions, beliefs, and threats, understanding the factors that influence vaccine hesitancy is essential for designing effective educational campaigns about the COVID-19 vaccination. 

## 2. Methodology

### 2.1. Criteria for Inclusion and Exclusion

There were three main inclusion criteria: (1) quantitative studies that used the HBM framework to examine relationships between HBM constructs and COVID-19 vaccine hesitancy and reported statistical tests of the relationships, (2) studies published in peer-reviewed journals, and (3) studies published in English between January 2020 and May 2022. We excluded qualitative studies, non-peer-reviewed studies, conference proceedings, case reports, and other grey literature. We also excluded a number of articles that mentioned vaccine hesitancy in the titles, articles not measuring vaccine hesitancy, or measured vaccine intention/acceptance rather than hesitancy. The reason is that vaccine hesitancy may not be a synonym for vaccine intention or acceptance. Vaccine hesitancy largely refers to a delay in the acceptance of vaccines despite the availability of vaccination services, but vaccine intention refers to the intention to take a vaccine when offered.

### 2.2. Search Strategy

This systematic review was performed according to the guidelines of Preferred Reporting Items for Systematic Reviews and Meta-Analyses (PRISMA) [9]. PubMed, Web of Science, Google Scholar, and Scopus databases were searched for articles on COVID-19 vaccine hesitancy. The search was conducted from 3 March 2022 to 15 May 2022. We conducted a comprehensive search of published literature from each of the four selected databases using the combinations of key terms and Boolean operators (see Table 1) such as “health belief model” or “HBM”, “vaccination hesitancy”, “vaccine hesitancy”, “COVID-19”, “corona virus”, “booster shot or dose”, and “SARS-CoV-2”.

**Figure 1:**
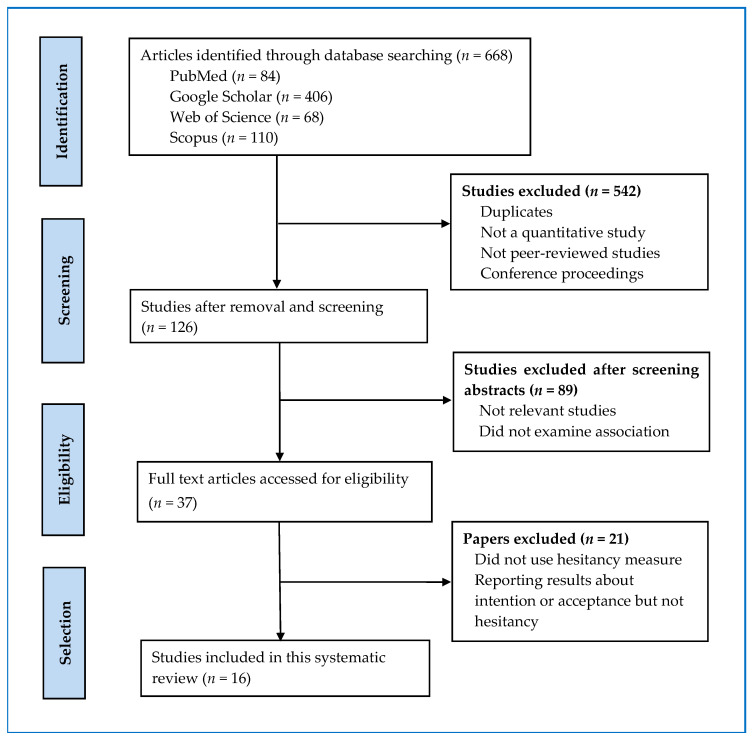
PRISMA flow diagram showing search strategy and study selection process.

The PRISMA diagram (Figure 1) illustrates the selection process and shows the reasons for exclusion. Initially, the titles and abstracts of all identified articles by a search were screened by three investigators independently. On the basis of the titles and abstracts, non-quantitative studies and the studies not applying the health belief model framework for predicting vaccine hesitancy were excluded. Full-text articles of eligible studies were obtained. These full-text articles were then evaluated to confirm if they reported necessary statistics on the relationship between HBM constructs and vaccine hesitancy.

As evident from Figure 1, the search process resulted in 668 studies, and out of them, 542 were removed for duplicates and not being quantitative and peer-reviewed journal articles. Of the remaining 126 studies, 89 were excluded as they were not relevant studies and did not examine the association between HBM constructs and COVID-19 vaccine hesitancy. The remaining 37 full-text articles were further assessed for eligibility. Of them, 21 articles were removed as they did not use a hesitancy measure or did not report required statistics, or reported results about vaccination intention or acceptance rather than vaccine hesitancy. The remaining 16 studies were found eligible for this review. 

### 2.3. Data Extraction and Analysis

The same three authors extracted data independently. The following information was extracted from each study: author’s name, data collection year, publication year, study objective, study design, participants, sample size, sampling method, measures, statistical analysis techniques, analytical tools, the country where the study was conducted, and hesitancy rate. We also extracted information on HBM factors associated with vaccine hesitancy i.e., susceptibility, severity, benefits, barriers, cues to action, self-efficacy, and modifying factors. The outcome variable was vaccine hesitancy. The extracted data were stored in a Microsoft Excel spreadsheet.

Data were analyzed using IBM SPSS Statistics 27. First, the characteristics of studies included in the review were summarized using frequencies and percentages. The average vaccination hesitancy rate was reported by country, continent, sample, and year of data collection.

## 3. Results

### 3.1. Characteristics of the Included Studies

Of the sixteen articles included in this study, eight articles were published in 2021 and eight in 2022 (see Table 2). Nine studies collected data in 2021, four in 2020, and two in 2020–2021. Eleven studies were conducted in Asia, three in North America, and two in Europe. Surprisingly, none of these studies was undertaken in South America and Australia. These studies represent nine countries with four from China, three from the United States, and two from Bangladesh. 

All studies were cross-sectional in design. The studies included in this review consisted of 30,242 respondents with a sample size of the studies ranging from 483 to 9153 (mean = 1890.13, SD = 2039.1). The study sample of eight studies were general adult populations with an age of 18 years and above. Other samples included healthcare workers, patients, students, pregnant women, and parents. All studies used non-random sampling. 

### 3.2. Vaccination Hesitancy Rate

Overall vaccination hesitancy rate for COVID-19 was 33.23% (95% CI 24.71–41.39%, SD = 17.35) ranging from 8.44% to 60.6% (see Table 2). Surprisingly, the highest vaccination hesitancy rate was reported in France (60.6%), followed by China (56.4%), South Korea (53.3%), Bangladesh (46.2%), and the USA (43.5%). The vaccination hesitancy rate was higher in Europe (42.68%) and Asia (33.49%) than in North America (25.97%). The vaccination hesitancy rate declined from 2020 (38.78%) to 2021 (33.59%). Vaccine hesitancy was highest among diabetes patients (56.4%), followed by general adult populations (36.9%), and students (27.53%). Vaccine hesitancy was lowest among healthcare workers (15%). 

### 3.3. HBM Constructs Associated with Vaccine Hesitancy

As shown in Table 3, sixteen studies used HBM as a theoretical framework and examined the relationships between HBM constructs and the COVID-19 vaccine hesitancy. All six HBM constructs were significantly associated with vaccine hesitancy. Perceived barriers and perceived benefits were the most common determinants that were significantly associated with vaccine hesitancy. Perceived benefits was inversely associated with COVID-19 vaccine hesitancy in twelve studies [6,11,12,13,15,16,17,18,20,22,23]. A positive association between perceived barriers and vaccine hesitancy was reported by twelve studies [6,11,12,13,15,16,17,18,19,20,22,24]. Perceived susceptibility was negatively correlated with COVID-19 vaccine hesitancy in eight studies [10,12,14,16,17,18,20,23]; however, Chen et al. [11] reported a positive correlation between perceived susceptibility and vaccine hesitancy. This means the participants were more likely to be vaccine-hesitant if they had a high perceived susceptibility to COVID-19. 

Other HBM components that are inversely related were cues to action in nine studies [6,10,11,16,17,19,21,22,24], perceived severity in six studies [14,16,17,19,23,24], and self-efficacy in one study [11]. Griva et al. [13] and Guillon and Kergall [6] found a negative effect of perceived threat (i.e., a combined measure of perceived susceptibility and severity) on vaccine hesitancy.

Individuals who afforded greater importance to cues to action from government, public health officials, and healthcare experts were also less likely to be hesitant [10]. A family member who got infected with Coronavirus and respondents who heard about the COVID-19 vaccine from social media (e.g., Facebook) or online news portals were less vaccine-hesitant [16]. The absence of perceived barriers, a high level of perceived benefits, and self-efficacy as well as an individual’s agreement with recommendations from authorities, friends, or family (cues to action) were negatively associated with COVID-19 vaccine hesitancy [11].

### 3.4. Modifying HBM Constructs Associated with Vaccine Hesitancy

The most common HBM modifying factor that was directly associated with COVID-19 vaccine hesitancy was gender in eight studies. The studies found that women were more likely to be COVID-19 vaccine-hesitant compared to men [6,10,11,12,13,20,21,23]. However, parental vaccine hesitancy for children vaccination was higher for males (father) [13].

In four studies, education was significantly correlated to vaccine hesitancy [12,15,20,24]; however, the results are conflicting. Interestingly, Lee and You [20] found that college students were more likely to be vaccine-hesitant, but other studies [12,15,24] reported that individuals with high school education or lower were more reluctant to get vaccinated against COVID-19. 

COVID-19 vaccine hesitancy was found to be influenced by age, but the results are inconsistent. For example, younger people, especially those under 30 years were more vaccine-hesitant [22,23]. Likewise, Du et al. [12] found that individuals who were below 45 years were more likely to hesitate in receiving a COVID-19 vaccine. However, Lee and You [20] found that individuals who were 50 years or older were more vaccine-hesitant.

Hesitancy against the COVID-19 vaccine differed significantly across geographical locations. For example, vaccine hesitancy was higher among people who lived in eastern China [12] and the Beijing area [21]. Likewise, the respondents living in the city corporation areas and Khulna regions of Bangladesh had more hesitancy [15].

Research has shown that occupation influences the COVID-19 vaccine hesitancy [11,22]. For example, compared to medical personnel, nonmedical personnel were more likely to be vaccine-hesitant [11]. Toth-Manikowski et al. [22] found that physicians were less likely to be vaccine-hesitant compared to non-physician samples such as nurses, administrative staff, and healthcare technicians. 

The participant’s income was found to be associated with vaccine hesitancy. However, the evidence is inconsistent. For example, Badr et al. [10] reported that higher-income individuals were less likely to be vaccine-hesitant, but Chen et al. [11] found just the opposite result. This means participants were more likely to be vaccine-hesitant if they had a higher income.

Race and ethnicity, marital status, employment, occupation, and perceived convenience were significantly associated with vaccine hesitancy. For example, Toth-Manikowski et al. [22] found that Black or African Americans were more likely to be hesitant to get vaccinated against the COVID-19. Badr et al. [10] found that married individuals who perceived vaccination as being convenient were less likely to be vaccine-hesitant but unemployed individuals were more likely to be vaccine-hesitant. Interestingly, nursing and health professions students were more likely to be vaccine-hesitant when compared to medical students [14].

Other significant modifying variables that directly influenced vaccine hesitancy were knowledge of disease or COVID-19 [10,12,15,17], prior diagnosis of COVID-19, history of flu vaccination [6,14,19-20,22], religion [15], political leaning [22], and nationality [19]. Individuals who more often were vaccinated when recommended by healthcare workers are less likely to be vaccine-hesitant [6]. 

## 4. Discussion and Implications

Despite the global effort of the vaccination drive, the studies included in this review reported a high rate of vaccine hesitancy against COVID-19. The vaccine hesitancy was higher among diabetes patients and adult populations as compared to students and healthcare workers. Thus, COVID-19 vaccine educational campaigns should be tailored to specific groups such as patients with chronic conditions and adult populations. 

This review contributes to the literature in three ways. First, this study analyzed the theoretical framework to examine the relationships between HBM constructs (perceived susceptibility, perceived severity, perceived benefits, perceived barriers, cues to action, and self-efficacy) and COVID-19 vaccine hesitancy. Second, it reports the prevalence of HBM modifying variables [25] such as demographic (e.g., age, gender, race, ethnicity, education, income, marital status), psychosocial (e.g., peer and reference group pressure), and structural variables (e.g., knowledge about a given disease, prior contact with the disease) that were significantly associated with vaccine hesitancy. As vaccine hesitancy is a complex multifaceted and dynamic social process that reflects multiple webs of influence, meaning, and logic [26], understanding the applicability of the HBM as a theoretical framework and the impacts of its constructs on vaccine hesitancy can be helpful to design tailored and targeted strategies to resolve it. Finally, this review not only reports the overall COVID-19 vaccine hesitancy rate but also identifies the occurrence of factors that influenced it. These results will be further broken down by data collection year, country, continent, and sample type.

As per our knowledge, this is the first systematic review of quantitative studies that used the HBM as the theoretical framework to examine the constructs of HBM contributing to the COVID-19 vaccine hesitancy. The results indicate that the HBM significantly predicted vaccine hesitancy against COVID-19. This is consistent with the findings of previous studies that used HBM as a framework to predict a broad range of health behaviors including vaccination [27], screening [28], and smoking [29] behaviors.

Findings from this investigation provide important insights for public health interventions to reduce vaccine hesitancy, which has been reported as a key factor that posts critical challenges for the success of COVID-19 immunization programs [7]. However, vaccine hesitancy is a complicated and multifaceted phenomenon and a dynamic social process that reflects multiple webs of influence, meaning, and logic [26]. This entails the existence of cognitive, psychological, socio-demographic, and cultural factors that contribute to vaccine hesitancy [30,31,32,33]. Thus, successful COVID-19 education and awareness campaigns require a solid understanding of the scale and determinants of vaccine hesitancy; so that tailored and targeted strategies can be developed [26]. 

The results suggest that the HBM can be useful in predicting and understanding the facilitators and barriers to COVID-19 vaccine hesitancy. Thus, HBM-based interventions and education programs can be effective in promoting COVID-19 vaccination and reducing vaccine hesitancy. Thus, it is recommended that primarily five dimensions of HBM, namely: perceived susceptibility, perceived severity, perceived benefits, perceived barriers, and cues to action should be part of such programs. This review reveals that perceived benefits and perceived barriers were the two most common HBM constructs that were significantly associated with COVID-19 vaccine hesitancy. While perceived benefits was inversely associated with vaccine hesitancy, perceived barriers was positively related to vaccine hesitancy. This is consistent with the HBM in general, which suggests that individuals are less likely to get vaccinated when they do not see a benefit from such behaviors and perceive obstacles to getting the COVID-19 vaccination. Thus, vaccine communication efforts to lower the perceived risk of vaccine side effects and heighten the perceived benefits of the vaccine are required [21].

Other HBM constructs that were frequently examined and negatively associated with vaccine hesitancy included perceived susceptibility, cues to action, perceived severity, and self-efficacy. This may indicate that COVID-19 vaccination promotion interventions or campaigns that suggest targeted populations who are at a greater risk of getting the virus and apprised of the seriousness of negative consequences if infected, may be effective in reducing vaccine hesitancy. Such campaigns or interventions can help people to overcome the barriers and enhance their willingness to get vaccinated. 

The results also suggest that cues to action are inversely associated with vaccine hesitancy. The most prevalent cues were the illness of family members, information from social media and online news portals, recommendations from healthcare workers, and advice from family or friends. These findings, therefore, emphasize the important role that social media, healthcare workers, family, and friends play in educating, persuading, and guiding individuals to be vaccinated against COVID-19.

This review also finds a number of HBM modifying factors such as demographic, psychosocial, and structural variables that were significantly associated with vaccine hesitancy. The most common modifying variable that was directly associated with COVID-19 vaccine hesitancy was gender, followed by education, age, geographical locations, occupation, income, race and ethnicity, employment, and marital status. Thus, to combat COVID-19 vaccine hesitancy, vaccine promotion campaigns should consider incorporating sociodemographic factors and designing targeted interventions based on the needs of diverse populations. The results suggest that sociodemographic factors, especially gender, education, and age are key to reducing vaccine hesitancy. Vaccine education initiatives should target women, younger, unmarried, unemployed, college students, individuals with low levels of education, nonmedical personnel, non-healthcare workers, and African Americans. Vaccine communication campaigns targeting unemployed and low-income people who are facing financial adversity can highlight that the vaccine is free of charge [10]. 

## 5. Limitations and Future Research

Although this systematic review provides important insights into the determinants of vaccine hesitancy, this study also has some limitations that should be addressed in future research. First, this study analyzed the literature that used the health belief model as the theoretical basis to examine the associations between HBM constructs and vaccine hesitancy. Future research can examine the applicability of other theories such as the theory of planned behavior, the theory of reasoned action, the protection motivation theory, and the information-motivation-behavioral skills model. Second, this review shows that most studies examined demographics HBM modifying variables. Future research should focus on other forms of HBM modifying factors such as psychosocial variables (e.g., peer and reference group pressure),structural variables (e.g., knowledge about a given disease, prior contact with the disease), and investigate their impacts on vaccine hesitancy. Third, although we performed a systematic search of articles using PRISMA guidelines, this review identified only sixteen studies conducted primarily in developed and emerging countries. Future research is thus required to assess the applicability of the HBM in predicting the vaccine hesitancy of different samples from developing countries; especially from South America, Africa, Asia, and the Middle East. Such studies will provide global evidence on the HBM factors influencing vaccine hesitancy. Fourth, as stated previously, several studies that examined the impacts of HBM factors on vaccine hesitancy used intention or acceptance measures to assess hesitancy. As the vaccine intention/acceptance may not be a synonym for vaccine hesitancy, future research should develop and use a hesitancy measure. Finally, the results of this review revealed inconsistent findings on the relationship between HBM modifying factors (gender, age, education, and income) and vaccine hesitancy. Future research is needed to shed light on such inconsistent findings.

## 6. Conclusions

To our knowledge, this paper represents the first systematic review of quantitative studies examining the association between HBM factors and COVID-19 vaccine hesitancy. The findings suggest that HBM provides a useful framework for explaining and predicting COVID-19 vaccine hesitancy. Thus, public awareness and educational programs aimed at reducing COVID-19 vaccine hesitancy should consider using HBM as a framework.

## Figures and Tables

**Table 1:** Key terms or Boolean operators used for search.

Search	Search Terms (Boolean Operators)
1	“health belief model” AND “vaccination hesitancy” AND “COVID-19”
2	“health belief model” AND “vaccination hesitancy” AND “coronavirus”
3	“health belief model” AND “vaccination hesitancy” AND “SARS-CoV-2”
4	“health belief model” AND “vaccine hesitancy” OR “vaccine hesitant”AND “COVID-19” “coronavirus” “SARS-CoV-2”
5	“health belief model” AND “booster” AND “COVID-19” “coronavirus” “SARS-CoV-2”

**Table 2:** Key characteristics of studies included in this systematic review.

Authors	Year of Publication	Journal	Country	Vaccine Hesitancy %	Sample	N
Guillon and Kergall [6]	2021	Public Health	France	60.6	adult general population	1146
Badr et al. [10]	2021	Vaccines	USA	43.5	adult general population	1208
Chen et al. [11]	2021	Journal of Medical Internet Research	China	44.3	adult general population	2531
Du et al. [12]	2021	Frontiers in Medicine	China	8.44	reproductive women	3011
Griva et al. [13]	2021	Vaccines	Singapore	9.9	adult general population	1623
Hosek et al. [14]	2022	Vaccines	USA	19.4	students	1030
Hossain et al. [15]	2021a	PLoS ONE	Bangladesh	46.2	adult general population	1497
Hossain et al. [16]	2021b	Frontiers in Public Health	Bangladesh	41.1	adult general population	1497
Huynh et al. [17]	2022	Postgraduate Medicine	Vietnam	26.2	parents	1015
Jain et al. [18]	2021	Epidemiology and Infection	India	10.6	students	1068
Le et al. [19]	2022	BMC Public Health	Vietnam	40.4	students	911
Lee and You [20]	2022	Journal of Medical Internet Research	South Korea	53.3	adult general population	1016
Rehati et al. [21]	2022	Vaccines	China	31.6	students	9153
Toth-Manikowski et al. [22]	2022	American Journal of Infection Control	USA	15	health care workers	1974
Walsh et al. [23]	2022	Acta Psychologica	Ireland, UK	24.75	adult general population	1079
Wang et al. [24]	2022	Vaccines	China	56.4	patients	483
				$\underline{X}$ = 33.23, SD = 17.35		$\underline{X}$ = 1890.13, SD = 2039.1

**Table 3:** Components of Health Belief Model Influencing COVID-19 Vaccine Hesitancy.

Authors and Year	Perceived Susceptibility	Perceived Severity	Perceived Benefits	Perceived Barriers	Cues to Action	Self Efficacy	Modifying Variables
Guillon and Kergall [6]			× (−)	× (+)	× (−)		Female (+)
Badr et al. [10]	× (−)				× (−)		Female (+) Higher Income (−) Unemployment (+) Marital status (−) Individuals who perceived vaccination as being convenient (−)
Chen et al. [11]	× (+)		× (−)	× (+)	× (−)	× (−)	Female (+) Higher income (+) Health status: Poor self-rated health (+) Occupation: Non-medical personnel (+)
Du et al. [12]	× (−)		× (−)	× (+)			Female (+) Region: Eastern China (+) Older people (over 45 years) (+) Lower than high school education level (+) Low score on knowledge of COVID-19 (+)
Griva et al. [13]			× (−)	× (+)			Female (+) Male (+) parental vaccine hesitancy for children Employed respondents Aged 31 to 40 years old Income between $5000 and $12,999 Absence of chronic illnesses (+) Living with people in poor health (−) Subjective norm (−) Moral norm (−) Perceived personal necessity of vaccination (−)
Hosek et al. [14]	× (−)	× (−)					Medical discipline History of COVID-19 infection (+)
Hossain et al. [15]			× (−)	× (+)			Geographic region Knowledge about the vaccine (−) Vaccination process (−) Negative attitudes towards the vaccine (+) Conspiracy beliefs towards COVID-19 vaccine (+) Country of origin
Hossain et al. [16]	× (−)	× (−)	× (−)	× (+)	× (−)		
Huynh et al. [17]	× (−)	× (−)	× (−)	× (+)	× (−)		Knowledge of COVID-19
Jain et al. [18]	× (−)		× (−)	× (+)			Lack of awareness regarding their eligibility for COVID-19 vaccination (+) Lack of trust in government agencies (+)
Le et al. [19]		× (−)		× (+)	× (−)		History of flu vaccination (−), Nationality (Vietnamese vs. Cambodian and Lao) (+) Major (pharmacy vs. physiotherapy (+)
Lee and You [20]	× (−)		× (−)	× (+)			Female (+) Age in 50s and age over 60s (+) Lower trust in government (+) History of flu vaccination (−) Seeking COVID-19 vaccine-related information via social media (+)
Rehati et al. [21]	× (−)				× (−)		Female (+) Geographic region History of flu vaccination (−) Higher COVID-19 vaccine price concerns (+) Convenience to vaccinate (−) Doctors’ recommendation to vaccinate (−) Lack of knowledge of COVID-19 (+)
Toth-Manikowski et al. [22]			× (−)	× (+)	× (−)		Age: Younger (+) Occupation: Non-physicians (+) Ethnicity: Black or African American (+) Political affiliation: Republican (+) Allergic to any vaccine component
Walsh et al. [23]	× (−), UK sample × (−), Irish sample	× (−), UK sample × (−), Irish sample	× (−), UK sample × (−), Irish sample				Women (+) Age under age 30 (+) Negative vaccination attitudes (+) Peer influence (−) Government influence (−) Civic responsibility (+)
Wang et al. [24]		× (−)		× (+)	× (−)		Education (High school) Disagreement with physicians’ view that vaccination can reduce SARS-CoV-2 infection risk (+) Disagreement with the statement that relatives’ vaccination status would influence participants’ vaccination decision (+)

## Data Availability

Data generated in this study is available by contacting the first author, Yam B. Limbu, if requested reasonably.
